# Low‐Dose Radiation Therapy in Alzheimer’s disease: An Interim Neurocognitive Analysis from a Multicenter Phase II Clinical Trial

**DOI:** 10.1002/alz.084076

**Published:** 2025-01-09

**Authors:** Byoung Hyuck Kim, Keun You Kim, Woo‐Yoon Park, Aryun Kim, Eun‐Hee Hong, Ji Young Kim, Seong‐Jun Cho, Hak Young Rhee, Weon Kuu Chung

**Affiliations:** ^1^ Seoul Metropolitan Government Seoul National University (SMG‐SNU) Boramae Medical Center, Seoul Korea, Republic of (South); ^2^ Chungbuk National University Hospital, Cheongju‐si, Chungcheongbuk‐do Korea, Republic of (South); ^3^ Korea Hydro & Nuclear Power Co Ltd., Seoul Korea, Republic of (South); ^4^ Kyunghee University Hospital at Gangdong, Seoul Korea, Republic of (South)

## Abstract

**Background:**

Recent preclinical studies have revealed a significant reduction in amyloid‐β plaques and pro‐inflammatory cytokines in Alzheimer’s disease (AD) mouse models following low‐dose radiation therapy (LDRT). This phase II, multicenter, prospective, single‐blinded, randomized controlled trial (NCT05635968, funding from Korea Hydro & Nuclear Power: Grant No. A21IP11) aims to investigate the efficacy and safety of whole‐brain LDRT in patients with AD.

**Method:**

Probable AD dementia patients (MMSE 13‐24 & CDR 0.5‐1) with evidence of amyloid pathology (confirmed by a positive 18F‐flutemetamol PET scan) were randomly assigned to one of three groups. They received a total of six fractions of LDRT: Group A with total 0 cGy (sham irradiation), Group B with 24 cGy, and Group C with 300 cGy. The effectiveness of LDRT was assessed through 18F‐flutemetamol PET, brain MRI and neurocognitive function tests at baseline, and at 6 and 12 months post‐LDRT. The primary endpoint was the change in the ADAS‐K‐cog score. The secondary endpoints included the changes in 18F‐flutemetamol PET, brain MRI, and scores of K‐MMSE, CDR, CGA‐NPI and K‐iADL.

**Result:**

A total of 15 patients (5 in each group) who completed 6 months post‐LDRT follow‐up were analyzed in this interim analysis. Baseline ADAS‐K‐cog (mean values in the order of group A, B, and C: 40.8 vs. 30.2 vs. 37.4, p = 0.111), CDR (1.0 vs 0.7 vs. 0.8, p = 0.141), K‐MMSE (19.6 vs. 22.4 vs. 19.2, p = 0.125) scores were not statistically different across the three groups. After 6 months, mean differences in cognitive test scores compared to baseline were 3.4 vs. 0.2 vs 0.4 (ADAS‐K‐cog, p = 0.369), 0.6 vs 0.0 vs 0.0 (CDR, p = 0.089), and ‐1.8 vs. 0.8 vs. 1.2 (K‐MMSE, p = 0.081). Compared to Group A, more subjects in experimental groups (B+C) showed improvement in K‐iADL (0/5 in Group A vs. 4/10 in Group B+C) and CGA‐NPI (0/5 in Group A vs. 5/10 in Group B+C). LDRT was well‐tolerated in all patients without any adverse events.

**Conclusion:**

Whole‐brain LDRT for patients with AD was tolerable and demonstrated potential benefits in neurocognitive function tests. Quantitative imaging analysis will follow, and final results with the planned follow‐up are awaited.

